# Pulse Wave Velocity at Early Adulthood: Breastfeeding and Nutrition during Pregnancy and Childhood

**DOI:** 10.1371/journal.pone.0152501

**Published:** 2016-04-13

**Authors:** Carolina Avila Vianna, Bernardo Lessa Horta, Denise Petrucci Gigante, Fernando Celso Lopes Fernandes de Barros

**Affiliations:** 1 Department of Social Medicine, Federal University of Pelotas, Pelotas, Rio Grande do Sul, Brazil; 2 Postgraduate Program in Health and Behavior, Catholic University of Pelotas, Pelotas, Rio Grande do Sul, Brazil; Centro Cardiologico Monzino IRCCS, ITALY

## Abstract

**Background:**

Pulse wave velocity (PWV) is an early marker of arterial stiffness. Low birthweight, infant feeding and childhood nutrition have been associated with cardiovascular disease in adulthood. In this study, we evaluated the association of PWV at 30 years of age with birth condition and childhood nutrition, among participants of the 1982 Pelotas birth cohort.

**Methods:**

In 1982, the hospital births in Pelotas, southern Brazil, were identified just after delivery. Those liveborn infants whose family lived in the urban area of the city were examined and have been prospectively followed. At 30 years of age, we tried to follow the whole cohort and PWV was assessed in 1576 participants.

**Results:**

Relative weight gain from 2 to 4 years was positively associated with PWV. Regarding nutritional status in childhood, PWV was higher among those whose weight-for-age z-score at 4 years was >1 standard deviation above the mean. On the other hand, height gain, birthweight and duration of breastfeeding were not associated with PWV.

**Conclusion:**

Relative weight gain after 2 years of age is associated with increased PWV, while birthweight and growth in the first two years of life were not associated. These results suggest that the relative increase of weight later in childhood is associated with higher cardiovascular risk.

## Introduction

Cardiovascular disease (CVD) is the leading cause of death worldwide. According to the World Health Organization (WHO), in 2008, 17.3 million deaths were due to CVD and it is estimated that this number will increase to 23.3 million by 2030 [1]. The traditional risk factors for CVD are hypertension, diabetes mellitus (DM), dyslipidemia, obesity and smoking. The occurrence of most cardiovascular diseases is closely related to atherosclerosis, which is characterized by the accumulation of fatty plaques (atheroma) within arteries [2].

Arterial stiffness increases with age and among young people may accelerate the atherosclerotic process, leading to several pathological conditions, such as decreased vascular compliance, increased systolic blood pressure, hypertrophy and fibrosis of the left ventricle, decreased coronary perfusion and endothelial dysfunction that leads to decreased production of nitric oxide, increasing smooth muscle tonus [3–8]. Pulse wave velocity (PWV) has been used to assess arterial stiffness. Each ventricular ejection generates a pulse pressure, which is propagated throughout the arterial tree. Pulse wave velocity is determined by the geometric and elastic properties of the arterial wall and increases with arterial stiffness [9]. Therefore, PWV is considered as an early marker of atherosclerosis and risk of CVD [10–11]. Indeed, PWV is higher among subjects who have experienced cardiovascular events [12–13]. With regard to the development of atherosclerosis, autopsy studies of children and young adults have identified fatty streaks in the coronary arteries, suggesting that the atherosclerotic process starts in early life [14–16].

With respect to the risk factors of cardiovascular diseases, evidence suggests that early exposures, such as intrauterine growth, birthweight, nutrition status in childhood and infant feeding, may program the development of metabolic cardiovascular risk factors [17–18]. On the other hand, few studies have evaluated the effect of early exposures on early markers of atherosclerosis, such as PWV [19–25]. In 1995, Martyn et al. evaluated the relationship between PWV in adulthood and measures of body size at birth. The PWV was higher among those with lower birthweight and smaller head and abdominal circumferences [19]. Mzayek et al. observed, in the Bogalusa study, that low birthweight was associated with higher PWV at 35 years of age [20]. On the other hand, Montgomery and colleagues found no association between low birth size and PWV [21], whereas Murray et al. reported that birthweight was positively associated with PWV in men and negatively in women in aorto-iliac and aorto-dorsalis pedis segments, but that in the aorto-radial segment the association was negative in both sexes [22].

In relation to breastfeeding and timing of introduction of solid foods, Jonge et al. reported that those children who had never been breastfed had higher PWV at 6 years of age, smaller left atrial diameter (LAD) and less left ventricular mass, whereas breastfeeding was not associated with blood pressure [23]. Tauzin et al. reported that PWV in early adulthood was higher among those subjects who were born at less than 32 weeks of gestational age [24]. In relation to nutrition, Tennant et al. found that adults who presented severe malnutrition in childhood (marasmus, kwashiorkor) had decreased cardiac output, PWV, increased diastolic pressure and increased systemic vascular resistance [25].

This study was aimed at assessing the association between PWV at 30 years of age and infant feeding and nutritional status during pregnancy and early childhood.

## Methods

In 1982, the maternity hospitals located in Pelotas were visited daily and all births identified. The 5914 liveborn infants whose families lived in the urban area were examined and their mothers interviewed. These individuals have been followed several times at different ages. Details of the methods of this cohort are available elsewhere [26,27]. Between June 2012 and February 2013, we tried to follow the entire cohort, using multiple search strategies and 3701 subjects were interviewed and examined in the research clinic.

In the 2012–13 visit, pulse wave velocity (PWV) was assessed using the Sphygmocor® system (Atcor Medical, Version 9.0, Sydney, Australia), a noninvasive device, which measures the PWV with a tonometric transducer. Participants were asked to refrain from smoking, drinking alcohol, caffeine, eating or physical activity at least 30 minutes before exams. A trained technician took the measurements, after 5 minutes of rest with the participants in the supine position in a quiet environment with controlled temperature (22–24°C). The tonometer was lightly pressed on the participant's skin, after palpation of the right carotid and femoral pulses, with simultaneous recording of the electrocardiogram for synchronization of carotid and femoral pulse wave times.

The PWV was calculated from measurements of pulse transit time and distance traveled by the pulse wave. Pulse transit time was estimated using the intersecting tangent foot-to-foot algorithm. The distance traveled by the pulse wave was measured using a flexible tape as the distance from the suprasternal notch to the femoral site of the pulse wave recording and the distance from the carotid site of the pulse wave recording and the suprasternal notch. The PWV was estimated as the distance between the measurement sites divided by the transit time delay between the femoral and carotid pulse wave [10].

In the 2012–13 visit, weight was measured using an electronic scale with a maximum capacity of 150 kg (Tanita®). Height was measured with a portable stadiometer with an accuracy of 0.1 cm.

Birthweight was assessed by the hospital staff using pediatric scales that were calibrated weekly by the research team. Gestational age was estimated from the last menstrual period. Birthweight according to gestational age z-score was calculated from the mean and standard deviation of birthweight based on gestational age and sex of the Williams’s reference population [28]. Detailed information about the duration of breastfeeding was collected in childhood (1984 and 1986). In the present analyses, we used the information obtained closest to the time of weaning.

Weight and length/height measures were transformed into weight-for-age or length/height-for-age z-scores using the WHO growth curves [29]. Conditional weight and length/height gain were used to estimate childhood growth; this measure indicates how much an individual measurement moves away from its previous path and from the population. The conditional growth is the residual of a linear regression. Conditional variables were generated by regressing current size (weight or length/height) on birthweight and earlier measures of weight and length/height, and standardized residuals were derived. To estimate conditional height, current length or height was regressed on previous weight and length. Therefore, conditional height at 2 years was estimated by regressing length-for-age z-scores at 2 years on birthweight. On the other hand, conditional relative weight was estimated from current length/height and previous measures of length/height and weight. Therefore, conditional relative weight at 2 years was derived by regressing weight at 2 years on birthweight and length at 2 years. Positive values indicate that the child grew faster than expected based on their previous growth and its population. Conditional growth in length is considered as the increase in length that is not predicted by previous length measurements; conditional growth in weight is considered as the increase in weight that is not predicted by previous weight or length measurements. Conditional variables were expressed in z-scores.

The analyses were performed using Stata 12® statistical package. Sex, race, maternal schooling, income, smoking during pregnancy and birthweight were considered as possible confounders. Analysis of variance (ANOVA) was used to compare means and multiple linear regression to adjust for confounding factors. In the linear regression, we estimated the p-value comparing the differences among the categories of the variable. Furthermore, we also assessed the p-value using the continuous exposure variable.

This study was approved by the research ethics committee of the Faculty of Medicine, Federal University of Pelotas, affiliated with the National Council on Ethics in Research of the Ministry of Health. All participants signed an informed consent.

## Results

In the 2012–13 visit, 3701 individuals were interviewed, and PWV was assessed in 1576 participants (27% of the original cohort). We were not able to measure the PWV in the remaining 2125 subjects due to operational problems. Table 1 shows that the proportion of subjects who had the PWV measured was independent of socioeconomic and demographic characteristics, breastfeeding duration and maternal smoking during pregnancy.

**Table 1:** Proportion of all subjects followed up to 30 years and those with pulse wave velocity measurement. The 1982 Pelotas (Brazil) Birth Cohort Study.

Variables	% subjects followed at 30 years with PWV data	% subjects followed at 30 years without PWV data
**Gender**		
Male	51.1	46.0
Female	48.9	54.0
**Skin colour**		
White	75.3	76.9
Non-white	24.7	23.1
**Family income (minimum wage**)		
≤1	19.0	20.2
1.1–3	50.6	48.4
3.1–6	19.1	20.0
6.1–10	5.9	6.1
>10	5.4	5.3
**Education (years)**		
0–4	6.7	5.7
5–8	19.9	20.0
9–11	29.4	30.5
≥12	44.0	43.8
**Breastfeeding duration (months)**		
<1	20.2	22.1
1–2.9	25.7	25.6
3–5.9	24.4	22.1
6–8.9	9.1	9.9
9–11.9	4.2	3.8
≥12	16.4	16.5
**Maternal smoking during pregnancy**		
Yes	37.9	32.8
No	62.1	67.2

PWV was lower among white individuals (P = 0.01). On the other hand, no association was observed for family income and education. (Table 2)

**Table 2:** Pulse wave velocity according to socioeconomic and demographic variables at 30 years of age. The 1982 Pelotas (Brazil) Birth Cohort Study.

Variables	N	Mean Pulse Wave Velocity in m/s (sd)
**Sex**		P = 0.06
Male	805	6.47(1.10)
Female	771	6.37 (1.08)
**Skin colour**		P = 0.01
White	1185	6.38 (1.07)
Non-white	389	6.54 (1.15)
**Family income (minimum wage)**		P = 0.21
≤1	299	6.36 (1.0)
1.1–3	794	6.46 (1.07)
3.1–6	299	6.42 (1.20
6.1–10	93	6.53 (1.05)
>10	84	6.21(1.31)
**Achieved schooling (years)**		P = 0.07
0–4	104	6.20 (0.85)
5–8	309	6.48 (1.12)
9–11	458	6.48 (1.02)
≥12	684	6.39 (1.16)

Table 3 shows that PWV was independent of birthweight and birthweight according to the gestational age z-score. Additionally, breastfeeding duration was not related to PWV. On the other hand, PWV was higher among those subjects whose weight-for-age z-score at 4 years of age was > 1 standard deviation above the mean. With regards to growth in childhood, relative weight gain from 2 to 4 years was positively associated with PWV, whereas there was no clear pattern for relative weight gain in the first two years, and the confidence intervals for each of the categories of relative weight gain included the null value. Length/height gain in the first 4 years was not associated with PWV.

**Table 3:** Pulse wave velocity according to pregnancy variables, breastfeeding, weight and height at 2 and 4 years of age.

Variables	N	Mean PWV m/s (sd)	Adjusted regression coefficient (95% CI)
**Birthweight (g)**		P = 0.98	P = 0.26^#^* P = 0.94^#^**
≥3500	499	6.43 (1.14)	0 (Reference)
3000–3499	583	6.42 (1.10)	-0.02 (-0.15;0.11)
2500–2999	375	6.43 (1.07)	-0.03 (-0.18;0.12)
<2500	119	6.38 (0.98)	-0.07 (-0.29;0.16)
**Birthweight for gestational age (z-score)**		P = 0.64	P = 0.37^#^* P = 0.59^#^**
< -1.28	177	6.36 (1.06)	0 (Reference)
-1.28–0	543	6.45 (1.05)	0.10 (-0.09;0.29)
>0	531	6.42 (1.16)	0.08 (-0.11;0.27)
**Breastfeeding duration (months)**		P = 0.52	P = 0.19^##^* P = 0.56^##^**
<1	311	6.46 (1.12)	0 (Reference)
1–2.9	393	6.35 (1.12)	-0.11(-0.28;0.05)
3–5.9	374	6.39 (1.05)	-0.07(-0.24;0.10)
6–8.9	139	6.52 (0.98)	0.06(-0.16;0.29)
9–11.9	64	6.48 (1.08)	-0.01(-0.29;0.36)
≥12	252	6.47 (1.17)	-0.01(-0.19;0.18)
**Weight for age (z-score) age of 2**		P = 0.13	P = 0.14^#^* P = 0.07^#^**
<-1	216	6.43 (1.04)	0 (Reference)
-1–0.99	955	6.40 (1.12)	-0.02(-0.19;0.15)
≥1	288	6.54 (1.08)	0.15(-0.05;0.35)
**Weight for age (z-score) age of 4**		P = 0.001	P = 0.005^#^* P = 0.001^#^**
<-1	241	6.40 (1.05)	0 (Reference)
-1–0.99	958	6.38 (1.07)	-0.003(-0.16;0.16)
≥1	227	6.67 (1.26)	0.31(0.11;0.52)
**Length for age (z-score) age of 2**		P = 0.09	P = 0.10^#^* P = 0.04^#^**
<-1	565	6.42 (1.05)	0 (Reference)
-1–0.99	786	6.41 (1.14)	0.03(-0.09;0.16)
≥1	108	6.65 (0.99)	0.29(0.06;0.53)
**Length for age (z-score) age of 4**		P = 0.006	P = 0.05^#^* P = 0.003^#^**
<-1	539	6.39 (1.03)	0 (Reference)
-1–0.99	795	6.42 (1.14)	0.04(-0.08;0.16)
≥1	89	6.78 (1.08)	0.43(0.18;0.68)
**Conditional height (age of 2**)		P = 0.47	P = 0.56^#^* P = 0.25^#^**
<0	535	6.41(1.11)	0 (Reference)
0.0001–1	358	6.43(1.08)	0.03(-0.12;0.19)
> 1	177	6.52(1.15)	0.16(-0.04;0.35)
**Conditional height (age of 4)**		P = 0.19	P = 0.13^#^* P = 0.22^#^**
<0	550	6.43(1.11)	0 (Reference)
0.0001–1	347	6.38(1.05)	-0.07(-0.22;0.08)
> 1	173	6.56(1.19)	0.11(-0.08;0.30)
**Conditional weight (age of 2)**		P = 0.03	P = 0.19^#^* P = 0.02^#^**
<0	561	6.44(1.12)	0 (Reference)
0.0001–1	335	6.33(0.99)	-0.12(-0.27;0.04)
> 1	174	6.61(1.25)	0.16(-0.03;0.35)
**Conditional weight (age of 4)**		P = 0.03	P = 0.02^#^* P = 0.01^#^**
<0	567	6.37(1.12)	0 (Reference
0.0001–1	353	6.44(1.04)	0.06(-0.09;0.21)
> 1	150	6.65(1.21)	0.27(0.07;0.47)

#Adjustment for sex, skin colour, education, family income and smoking during pregnancy.

## Adjustment for sex, skin colour, education, family income, smoking during pregnancy and birth weight.

* P-value based on continuous variables.

** P-value based on categorical variables.

## Discussion

In this cohort that has been prospectively followed since birth, in a southern Brazilian city, we observed that relative weight gain from 2 to 4 years of age was associated with increased PWV, whereas birthweight, relative weight gain in the first two years of life, and linear growth (length/height gain) in childhood were not associated with PWV. Furthermore we did not observe any programming effect of breastfeeding on PWV at 30 years of age.

Information on birthweight, gestational age, breastfeeding duration and nutritional status was collected close to the time of its occurrence, reducing the possibility of misclassification. In addition, the absence of any trend in the association between breastfeeding and PWV suggests that it is unlikely that measurement error underestimated an association. By the same token, confounding variables were measured in early childhood, reducing the chance of residual confounding. On the other hand, PWV was not assessed for all subjects who were followed at the age of 30, but PWV examination was independent of socioeconomic status, breastfeeding duration and nutritional status, suggesting that selection bias is unlikely. On the other hand, we were not able to adjust the conditional growth estimates to birth length because information on this variable had not been collected in the perinatal study.

With regard to the long-term effects of breastfeeding on the risk of cardiovascular disease, some studies have observed that breastfeeding is inversely associated with cardiovascular risk factors [23,30], whereas others have not observed such an association [31–36]. Most studies that observed a protective effect of breastfeeding had been carried out in high-income countries, where breastfeeding is more common among wealthier subjects. Therefore, the observed associations could be due to residual confounding. Brion et al. found in the Pelotas (Brazil) birth cohort, where breastfeeding is not associated with socioeconomic status, that breastfeeding was not associated with prevalence of obesity and blood pressure [37], whereas in the ALSPAC cohort [38], where breastfeeding is positively associated with socioeconomic status, an association was observed. Similarly, in the PROBIT study, blood pressure was similar in the both intervention and control groups [39]. A recently published meta-analysis, found that breastfeeding protects against overweight/obesity and type-2 diabetes, but was not associated with blood pressure and total cholesterol [40]. As our study evaluated young adults without chronic diseases, it is not surprising that we have not found an association between breastfeeding and PWV.

With respect to childhood growth, it has been reported that growth in early childhood is not associated with an increased risk, whereas weight gain in late childhood is positively associated with cardiovascular risk factors [41–47]. Furthermore, Menezes et al. observed that relative weight gain and not linear growth is associated with higher risk [48]. Therefore, our findings reinforce the evidence that a relative increase in weight gain later in childhood is positively associated with a marker of atherosclerosis in early adulthood. Therefore, only early weight gain should be promoted.

## References

[1] Alwan A, Armstrong T, Bettcher D, Branca F, Chisholm D, Ezzati M et al Global Status Report on noncommunicable diseases WHO 2010;1–162. http://www.who.int/nmh/publications/ncd_report_full_en.pdf

[2] Braunwald E, Bonow RO, Libby P, Zipes DP. Braunwald´s Heart Disease: a textbook of cardiovascular medicine, 7th edition, Elsevier 2005.

[3] Farra DJ, Bond MG, Riley WA, Sawyer JK. Anatomic correlates of aortic pulse wave velocity and carotid artery elasticity during atherosclerosis progression and regression in monkeys. Circulation.1991, 83:1754–1763 10.1161/01.CIR.83.5.1754 2022028

[4] London GM, Marchais SJ, Guerin AP, Metivier F, Pannier B. Cardiac hypertrophy and arterial alterations in end-stage renal disease: hemodynamic factors. Kidney International Supplement.1993; 41:S42–S49. 8320946

[5] O’Rourke MF, Safar ME. Relationship between aortic stiffening and microvascular disease in brain and kidney: cause and logic of therapy. Hypertension. 2005; 46:200–204. 15911742 10.1161/01.HYP.0000168052.00426.65

[6] Ohtsuka S, Kakihana M, Watanabe H, Sugishita Y. Chronically decreased aortic distensibility causes deterioration of coronary perfusion during increased left ventricular contraction. Journal of the American College of Cardiology. 1994;24:1406–1414. 7930267 10.1016/0735-1097(94)90127-9

[7] Lacolley P, Challande P, Osborne-Pellegrin M, Regnault V. Genetics and pathophysiology of arterial stiffness. Cardiovascular Research. 2009; 81:637–648. 10.1093/cvr/cvn353 19098299

[8] Zieman SJ, Melenovsky V, Kass DA. Mechanisms, Pathophysiology, and Therapy of Arterial Stiffness. Arterioscler Thromb Vasc Biol. 2005;25:932–943. 15731494 10.1161/01.ATV.0000160548.78317.29

[9] Tomlinson LA. Methods for assessing arterial stiffness: technical considerations. Curr Opin Nephrol Hypertens 2012, 21:000–000 10.1097/MNH.0b013e32835856e3 22895411

[10] Laurent S, Cockcroft J, Bortel LV, Boutouyrie P, Giannattasio C, Hayoz D et al Expert consensus document on arterial stiffness: methodological issues and clinical applications. European Heart Journal. 2006;27:2588–2605. 17000623 10.1093/eurheartj/ehl254

[11] Mitchell GF, Hwang SJ, Vasan RS, Larson MG, Pencina MJ, Hamburg NM et al Arterial stiffness and cardiovascular events: the Framingham heart study. Circulation. 2010; 121:505–11. 10.1161/CIRCULATIONAHA.109.886655 20083680 PMC2836717

[12] Maldonado J, Pereira T, Polónia J, Silva JA, Morais J, Marques M. Arterial stiffness predicts cardiovascular outcome in a low-to-moderate cardiovascular risk population: the EDIVA (Estudo de Distensibilidade Vascular) Project. Journal of Hypertension 2011:4(29)669–75. 10.1097/HJH.0b013e3283432063 21252699

[13] Boutouyrie P, Tropeano AI, Asmar R, Gautier I, Benetos A, Lacolley P et al Aortic Stiffness Is an Independent Predictor of Primary Coronary Events in Hypertensive Patients—A Longitudinal Study. Hypertension. 2002;39:10–15 10.1161/hy0102.099031 11799071

[14] Newman WP III, Wattigney W, Berenson GS. Autopsy studies in United States children and adolescents. Relationship of risk factor to atherosclerotic lesions. Annals of the New York Academy of Sciences. 1991;623:16–25. 2042824 10.1111/j.1749-6632.1991.tb43715.x

[15] Mendis S, Nordet P, Fernandez-Britto JE, Sternby N. Atherosclerosis in children and young adults: An overview of the World Health Organization and International Society and Federation of Cardiology study on Pathobiological Determinants of Atherosclerosis in Youth study (1985–1995). Prevention and Control. 2005;1:3–15.

[16] Tracy RE, Newman WP, Wattigneyb WA, Srinivasanb SR, Stronga JP, Berenson GS. Histologic features of atherosclerosis and hypertension from autopsies of young individuals in a defined geographic population: the Bogalusa Heart Study. Atherosclerosis. 1995;116(2):163–79. 7575772 10.1016/0021-9150(95)05525-2

[17] Barker DJP. Fetal origins of coronary heart disease. BMJ 1995; 311:171–4. 7613432 10.1136/bmj.311.6998.171 PMC2550226

[18] Risnes KR, Vatten LJ, Baker JL, Jameson K, Sovio U, Kajantie E et al Birthweight and mortality in adulthood: a systematic review and meta-analysis. International Journal of Epidemiology 2011;40:647–661 10.1093/ije/dyq267 21324938

[19] Martyn CN, Barker DJP, Jespersen S, Greenwald S, Osmond C, Berry C. Growth in utero, adult blood pressure, and arterial compliance. British Heart Journal 1995;73:116–121. 7696018 10.1136/hrt.73.2.116 PMC483775

[20] Mzayek F, Sherwin R, Hughes J, Hassig S, Srinivasan S, Chen W et al The association of birth weight with arterial stiffness at mid-adulthood: the Bogalusa Heart Study. Journal of epidemiology and community health. 2009;63(9):729–33. 10.1136/jech.2008.084475 19429574

[21] Montgomery AA, Ben-Shlomo Y, McCarthy A, Davies D, Elwood P, Smith GD. Birth size and arterial compliance in young adults. Lancet. 2000 6 17;355(9221):2136–7. 10902632 10.1016/S0140-6736(00)02385-0

[22] Murray LJ, Gallagher AM, Boreham CAG, Savage M, Smith GD. Sex specific difference in the relation between birth weight and arterial compliance in young adults: The Young Hearts Project. J Epidemiol Community Health 2001;55:665–666. 11511646 10.1136/jech.55.9.665 PMC1731966

[23] Jonge LL, Langhout MA, Taal HR, Franco OH, Raat H, Hofman A et al Infant Feeding Patterns Are Associated with Cardiovascular Structures and Function in Childhood. American Society for Nutrition. 2013 10.3945/jn.113.174326 24089417

[24] Tauzin L, Rossi P, Grosse C, Boussuges A, Frances Y, Tsimaratos M et al Increased systemic blood pressure and arterial stiffness in young adults born prematurely. Journal of Developmental Origins of Health and Disease, 2014 10.1017/S2040174414000385 25154472

[25] Tennant IA, Barnett AT, Thompson DS, Kips J, Boyne MS, Chung EE et al Impaired cardiovascular structure and function in adult survivors of severe acute malnutrition. Hypertension. 2014 9;64(3):664–71. 10.1161/HYPERTENSIONAHA.114.03230 Epub 2014 Jun 30. 24980666

[26] Horta BL, Gigante DP, Gonçalves H, Motta JVS, de Mola CL, Oliveira IO et al Cohort Profile Update: The 1982 Pelotas (Brazil) Birth Cohort Study. International Journal of Epidemiology, 2015, 1–6 10.1093/ije/dyv017 PMC4469796 25733577

[27] Barros FC, Victora CG, Horta BL, Gigante DP. Metodologia do estudo da coorte de nascimentos de 1982 a 2004–5, Pelotas, RS. Rev Saude Publ. 2008;42:7–15. 10.1590/s0034-89102008000900003 PMC2671798 19142340

[28] Williams RL, Creasy RK, Cunnigham GC, Hawes WE, Norris FD, Tashiro M. Fetal growth and perinatal viability in California. Obstetrics and gynecology. 1982;59:624–32. 7070736

[29] de Onis M, Onyango A, Borghi E, Siyam A, Pinol A, Garza C et al WHO Multicentre Growth Reference Study Group. WHO Child Growth Standards: Length/height-for-age, weight-for-age, weight-for-length, weight-for-height and body mass index-for-age: Methods and development. World Health Organization. 2006. 312 pages.

[30] Singhal A, Lucas A. Early origins of cardiovascular disease: is there a unifying hypothesis? Lancet. 2004;363:1642e5. 5 15145640 10.1016/S0140-6736(04)16210-7

[31] Owen CG, Whincup PH, Gilg JA, Cook DG. Effect of breastfeeding in infancy on blood pressure in later life: systematic review and meta- analysis. BMJ. 2003;327:1189–95. 14630752 10.1136/bmj.327.7425.1189 PMC274051

[32] Martin RM, Gunnell D, Smith GD. Breastfeeding in infancy and blood pressure in later life: systematic review and meta-analysis. American Journal of Epidemiology. 2005;161:15–26. 15615909 10.1093/aje/kwh338

[33] Leeson CP, Kattenhorn M, Deanfield JE, Lucas A. Duration of breastfeeding and arterial distensibility in early adult life: population based study. BMJ. 2001;322:643–7. 11250848 10.1136/bmj.322.7287.643 PMC26543

[34] Schack-Nielsen L, Molgaard C, Larsen D, Martyn C, Michaelsen KF. Arterial stiffness in 10-year-old children: current and early determinants. British Journal of Nutrition. 2005;94:1004–11. 16351780 10.1079/bjn20051518

[35] Järvisalo MJ, Hutri-Kahonen N, Juonala M, Mikkila V, Rasanen L, Lehtimaki T et al Breastfeeding in infancy and arterial endothelial function later in life: the Cardiovascular Risk in Young Finns Study. European Journal of Clinical Nutrition. 2009;63:640–5. 10.1038/ejcn.2008.17 18285807

[36] Salvi P, Revera M, Joly L, Reusz G, Iaia M, Benkhedda S et al Role of birth weight and postnatal growth on pulse wave velocity in teenagers. The Journal of adolescent health. 2012 10.1016/j.jadohealth.2012.01.009 22999838

[37] Brion MJ, Lawlor DA, Matijasevich A, Horta B, Anselmi L, Araújo CL et al What are the causal effects of breastfeeding on IQ, obesity and blood pressure? Evidence from comparing high-income with middle-income cohorts. International Journal of Epidemiology. 2011 10.1093/ije/dyr020 PMC3147072 21349903

[38] Toschke AM, Martin RM, von Kries R, Wells J, Smith GD, Ness AR. Infant feeding method and obesity: body mass index and dual-energy X-ray absorptiometry measurements at 9–10 y of age from the Avon Longitudinal Study of Parents and Children (ALSPAC). American Journal of Clinical Nutrition. 2007;85(6):1578–85. 17556696 10.1093/ajcn/85.6.1578

[39] Kramer MS, Chalmers B, Hodnett ED, Sevkovskaya Z, Dzikovich I, Shapiro S et al PROBIT Study Group (Promotion of Breastfeeding Intervention Trial). Promotion of Breastfeeding Intervention Trial (PROBIT): a randomized trial in the Republic of Belarus. JAMA. 2001;285(4):413–20 11242425 10.1001/jama.285.4.413

[40] Horta BL, de Mola CL, Victora CG. Long-term consequences of breastfeeding on cholesterol, obesity, systolic blood pressure, and type-2 diabetes: systematic review and meta-analysis. Acta Paediatr. 2015 7 20 10.1111/apa.13133 26192560

[41] Ong KK, Loos RJF. Rapid infancy weight gain and subsequent obesity: Systematic reviews and hopeful suggestions. Acta Paediatrica. 2006;95:904e8. 6 16882560 10.1080/08035250600719754

[42] Sachdev HS, Fall CH, Osmond C, Lakshmy R, Dey Biswas SK, Leary SD et al Anthropometric indicators of body composition in young adults: relation to size at birth and serial measurements of body mass index in childhood in the New Delhi birth cohort. American Journal of Clinical Nutrition. 2005;82:456e66. 16087993 10.1093/ajcn.82.2.456

[43] Barker DJ, Winter PD, Osmond C, Margetts B, Simmonds SJ. Weight in infancy and death from ischaemic heart disease. Lancet. 1989;2:577e80. 2570282 10.1016/s0140-6736(89)90710-1

[44] Lucas A, Fewtrell MS, Cole TJ. Fetal origins of adult disease-the hypothesis revisited. BMJ. 1999;319:245e9. 10417093 10.1136/bmj.319.7204.245 PMC1116334

[45] Stettler N, Kumanyika SK, Katz SH, Zemel BS, Stallings VA. Rapid weight gain during infancy and obesity in young adulthood in a cohort of African Americans. American Journal of Clinical Nutrition. 2003;77:1374e8. 12791612 10.1093/ajcn/77.6.1374

[46] Adair LS, Martorell R, Stein AD, Hallal PC, Sachdev HS, Prabhakaran D et al Size at birth, weight gain in infancy and childhood, and adult blood pressure in 5 low- and middle-income-country cohorts: when does weight gain matter? American Journal of Clinical Nutrition. 2009;89:1383e92. 19297457 10.3945/ajcn.2008.27139 PMC2720838

[47] Kelishadi R, Haghdoost AA, Jamshidi F, Aliramezany M, Moosazadeh M. Low birthweight or rapid catch-up growth: which is more associated with cardiovascular disease and its risk factors in later life? A systematic review and cryptanalysis. Paediatrics and International Child Health. 2014 18:2046905514Y0000000136. 10.1179/2046905514Y.0000000136 25034799

[48] Menezes AM, Hallal PC, Dumith SC, Matijasevich AM, Araújo CL, Yudkin J et al Adolescent blood pressure, body mass index and skin folds: sorting out the effects of early weight and length gains. Journal of Epidemiology and Community Health. 2012 10.1136/jech.2010.124842 PMC3245895 21325148

